# Improving Care for People With Aphasia: Communication Training for Healthcare Providers

**DOI:** 10.1111/1460-6984.70208

**Published:** 2026-02-13

**Authors:** Carolina Maurício, Pedro Sá‐Couto, Isabel Monteiro da Costa, Maria Assunção C. Matos

**Affiliations:** ^1^ School of Health Sciences (ESSUA) University of Aveiro Aveiro Portugal; ^2^ Center for Research and Development in Mathematics and Applications (CIDMA) Department of Mathematics (DMAT) University of Aveiro Aveiro Portugal; ^3^ RISE‐ Health, School of Health University of Aveiro Aveiro Portugal

**Keywords:** aphasia, communication, communicative training, healthcare professionals, Supported Conversation for Adults with Aphasia (SCA)

## Abstract

**Introduction:**

Communication barriers significantly impact the quality of care for people with aphasia (PWA). To address this, training healthcare professionals (HCPs) who interact with PWA is essential. The Health Professionals and Aphasia Questionnaire (HPAQ) assess the effectiveness of such training by measuring changes from pre‐ to post‐intervention.

**Objective:**

This study aimed to translate and validate the HPAQ into European Portuguese (HPAQ‐EP) and to analyse the effectiveness of the *Communicative Training in Aphasia* program by examining changes in HCPs’ communication skills at a clinical centre in Portugal using the newly developed HPAQ‐EP.

**Methods:**

A pre‐post intervention study was conducted in two phases. Phase 1 involved translating the HPAQ‐EP and validating it through expert panel review, in accordance with international guidelines. Content validity was assessed using the Content Validity Index (CVI). In Phase 2, the HPAQ‐EP was administered to HCPs at a clinical centre before and after a training session based on the Supported Conversation for Adults with Aphasia (SCA) approach.

**Results:**

The HPAQ‐EP demonstrated strong content validity (CVI > 0.80) and good internal consistency (*α* = 0.912), comparable to the original tool. Following implementation of the *Communicative Training in Aphasia* program (*n* = 23), significant improvements were observed, as reported by the HPAQ‐EP, in items related to ‘Knowledge’, ‘Skills’, ’Attitudes and Emotions’ (items 8 and 9) and ‘Environment (item 14). Participants found the aphasia simulation the most challenging part and it was particularly insightful for understanding the lived experience of PWA.

**Conclusions:**

This study shows that targeted communication training significantly enhances HCPs’ readiness to engage with PWA, highlighting its clinical value. The findings also confirm the HPAQ‐EP as a valid and reliable tool for assessing HCPs’ attitudes toward aphasia and for guiding the implementation of such training. Future efforts should emphasise long‐term follow‐up and broader implementation across healthcare settings.

**WHAT THIS PAPER ADDS:**

*What is already known on this subject*

- PWA often experience communication barriers that negatively affect their healthcare experiences and outcomes. HCPs frequently lack the specific training needed to effectively communicate with PWA, leading to frustration, miscommunication, increased medical errors and reduced patient involvement in their healthcare process and decisions. Communication Partner Training (CPT), particularly SCA, is an evidence‐based approach shown to improve HCPs communication skills. In Portugal, there is a lack of validated tools to assess the impact of such training and few interventions have targeted HCPs directly within clinical settings.

*What does this study adds to existing knowledge*

- The HPAQ was translated and validated into European Portuguese (HPAQ‐EP), demonstrating high content validity and internal consistency. It was used to evaluate the effectiveness of a theoretical‐practical *Communicative Training in Aphasia* program for HCPs. Post‐training results showed statistically significant improvements in knowledge, communication skills and attitudes towards PWA. Despite its limitations, this study offers a culturally adapted and psychometrically robust tool for Portuguese‐speaking HCPs, while also providing evidence of the short‐term effectiveness of communication training for multidisciplinary teams working with PWA in clinical settings.

*What are the potential or actual clinical implications of this work?*

- The findings support integrating targeted communication training into routine professional development for HCPs who interact with PWA. By improving HCPs knowledge, confidence and communicative strategies, such training fosters more inclusive, patient‐centred care and reduces the risk of marginalisation and medical errors. The validated HPAQ‐EP enables ongoing evaluation of training impact in Portuguese‐speaking settings. Broader implementation and long‐term follow‐up are recommended to maximise benefits and promote sustainable changes in clinical practice. This study advocates for the inclusion of aphasia‐specific communication modules in healthcare curricula to better prepare future HCPs.

## 1 Introduction

Aphasia is a communication disorder caused by an acquired language impairment, affecting social participation and, consequently, the quality of life of the person with aphasia and their relationships with family and friends (Fotiadou et al. 2014; Papathanasiou 2022). People with aphasia (PWA) may experience difficulties expressing themselves, understanding what others say and/or reading and writing. They may also struggle with understanding and/or using gestures (Kagan and Simmons‐Mackie 2013).

The leading cause of aphasia is stroke, with 20%–40% of cases resulting in acute aphasia. Other potential causes include brain tumour, head injuries, infections or other brain injuries (Simmons‐Mackie and Kagan 2007). When aphasia occurs as part of a degenerative disease, it is called Primary Progressive Aphasia (Rogalski and Khayum 2018).

Despite their language difficulties, many PWA retain intact cognitive and social capacities. However, these abilities are frequently overlooked in healthcare settings, where communication challenges often hinder their active involvement in care planning and decision‐making (Cameron et al. 2018). Research consistently shows that individuals with communication disorders, including aphasia, are at an increased risk of marginalisation within clinical environments. They are more likely to experience adverse outcomes (Bartlett et al. 2008), receive suboptimal care and be excluded from critical discussions about their diagnosis, treatment options and rehabilitation goals. Interactions are often limited to directive, one‐way communication, depriving patients of opportunities to express preferences, ask questions or fully engage in their care (Carragher et al. 2024; Hemsley et al. 2013).

A significant contributor to these communication inequities is the lack of formal training among healthcare professionals (HCPs) in how to interact effectively with PWA. Although HCPs may have general awareness of aphasia, they frequently lack practical skills and confidence in adapting their communication style to meet the needs of this population (Hur and Kang 2022). Recent investigations underscore the role of social and physical environments in aphasia experiences, showing that accessible communication via partner training improves access to patient‐centred care and adherence to clinical decision‐making (Cruice et al. 2018). Consequently, HCPs may experience frustration, resort to avoidance strategies (Carragher et al. 2021), or default to communicating primarily with family members rather than with the patients themselves (Burns et al. 2015). This undermines patient‐centred care and can compromise therapeutic outcomes.

To address these challenges, Communication Partner Training (CPT) has emerged as an evidence‐based approach designed to improve communication between PWA and their conversational partners, including HCPs, family members and volunteers. CPT typically incorporates education about aphasia, identification of communication strategies, structured practice and constructive feedback (Cruice et al. 2018; Kagan et al. 2001; Simmons‐Mackie et al. 2010; 2016). These interventions can be delivered individually, in dyads, or in groups and may or may not include direct participation from PWA (Kagan et al. 2001). A growing body of evidence demonstrates that CPT improves HCPs’ knowledge, confidence and communication behaviours, while also supporting the recognition of PWA's preserved competencies and promoting their participation in healthcare decisions (Cruice et al. 2018; Kagan et al. 2001; Simmons‐Mackie et al. 2010). Recent systematic reviews confirm that CPT consistently enhances partner communication skills and PWA participation, particularly in chronic contexts, yet highlight implementation variability, essential component specification and adaptation needs for complex settings such as hospitals (Cruice et al. 2018; Simmons‐Mackie et al. 2016).

The SCA is a widely recognised CPT method designed to facilitate communication with PWA through a variety of evidence‐based techniques. Its purpose is to support individuals with communication impairments in expressing their thoughts, opinions and emotions in ways that foster a sense of being valued and understood. By applying this method, communication partners, such as physicians, nurses, family members, friends and other HCPs, work to minimise communication barriers and support the reintegration of individuals with aphasia into everyday interactions (Kagan, 1998; Simmons‐Mackie et al. 2016).

Evaluating the effectiveness of CPT interventions requires appropriate outcome measures. Self‐report tools are often considered practical and feasible in both clinical and research contexts. The HPAQ was developed to assess HCPs’ perceptions and behaviours related to communicating with PWA and serves as a reliable and valid measure for monitoring the impact of training initiatives (Jensen et al. 2022).

In Portugal, few studies focused on the direct training of communication partners dealing with PWA (Matos et al. 2025). Ramos investigated the communication challenges faced by HCPs when providing care to PWA and concluded that awareness‐raising initiatives and training interventions should be developed for professionals working with this population to broaden their range of communicative strategies (Barros 2016; Ramos and Vital 2012). Pereira emphasised that, although HCPs need to adopt communication strategies when facing communication barriers, there is a lack of specific knowledge or training to ensure their practical use. In response, a communication strategies program was implemented within a team of HCPs, leading to an increase in both the number and frequency of communicative strategy use (Pereira 2015). According to Santos, most primary care HCPs in Portugal acknowledge that they lack sufficient training to develop effective communication skills in healthcare settings. Therefore, it is essential to implement a theoretical‐practical training program to improve the quality of care provided. Equally important is ensuring that the tools used to evaluate the effectiveness of such programs are both valid and reliable (Santos 2022).

As far as we know, no self‐reported outcome measure in Portuguese exists for HCPs who interact with PWA. This study aims to address this gap by translating and validating the HPAQ into European Portuguese (HPAQ‐EP) and by examining its utility in assessing the effectiveness of a structured, theoretical–practical *Communicative Training in Aphasia* program delivered to a team of HCPs at a clinical centre in Portugal. The ultimate goal is to enhance clinical communication practices and contribute to more equitable, patient‐centred care for individuals living with aphasia.

## 2 Methods and Procedures

### 2.1 Study Design

This study was conducted in two phases: Phase 1—Translation and content validation of the HPAQ‐EP; and Phase 2—Implementation of the *Communicative Training in Aphasia*, a theoretical‐practical program conducted in a workshop format with the specified team, focusing on strategies to facilitate communication with PWA.

The HPAQ‐EP was administered at both pre‐ and post‐training stages to statistically measure the training's impact on the team. Thus, the proposed study is an educational development project that incorporates quantitative variables and a prospective approach. Accordingly, it is characterised as a methodological study in Phase 1 and as an exploratory, prospective and correlational study in Phase 2 (Fortin 2009).

### 2.2 Formal and Ethical Procedures

This study was conducted within a military health institution, and all requisite authorisations were obtained in accordance with the established chain of command. Initial approval was obtained from the Head of the Rehabilitation Service, where the lead researcher was based. Subsequently, the Head of the Education Division formally authorised and granted the request via email. Finally, permission to involve participants from the Medical Nursing Service and the Mental Health Service was secured from the respective service heads. Ethical approval was also obtained from the institutional review board of the Portuguese National Republican Guard (PGNR) Clinical Center in Lisbon and relevant national and international guidelines, and all procedures complied with applicable data protection regulations.

## 3 Informed Consent and Participant Confidentiality

All potential participants received detailed information about the study's purpose, procedures, risks and benefits and provided written informed consent before enrolment. To maintain anonymity while enabling linkage of pre‐ and post‐training responses, each participant was assigned a unique six‐digit code composed of: (1) the first two digits of their citizen‐card number, (2) the first two digits of their tax‐identification number and (3) the final two digits of their mobile‐phone number. This coding scheme preserved confidentiality while allowing longitudinal data matching. Data handling and storage complied with institutional data protection policies throughout the study.

To facilitate comprehension and readability, the study's phases will be described individually.

## 4 Phase 1: Translation and Validation of the HPAQ

### 4.1 Procedures

#### 4.1.1 Instrument Selection

To assess the impact of the training intervention, the research team reviewed some candidate instruments, namely the Aphasia Attitudes, Strategies and Knowledge (AASK) (Power et al. 2020), the Measure of Skill in Supported Conversation (MSC) and the Measure of Participation in Conversation (MPC) (Kagan et al. 2004) and the HPAQ (Jensen et al. 2022). After individual review of each instrument and subsequent discussion among all team members, the HPAQ was chosen. No pre‐determined criteria were defined. The decision was based on its demonstrated applicability in clinical settings, its inclusion of diverse health professional roles with relevant field experience, its focus on constructs aligned with the Clinical Centre's context, as well as the goals of the study. Following selection, permission to translate and validate the HPAQ‐EP was requested from and granted by the instrument's original author via email.

#### 4.1.2 Translation and Validation Process

Translation and cross‐cultural adaptation procedures adhered to standard guidelines to ensure semantic and conceptual equivalence. The translation and validation process of the instrument was carried out in accordance with Coluci's protocol (Alexandre and Coluci 2011), which includes the following phases: initial translation, synthesis, expert review and pre‐testing. During this cultural adaptation process, it was also ensured that the experts were bilingual and specialists in the area of focus.

The translation of the instrument into EP was carried out by three speech‐language therapists (SLTs). Two of them independently translated the HPAQ from English to EP, while the third reviewed and compared the two versions to produce a consensus version. An expert panel evaluated this consensus version. Once selected, each expert received, via email, a demographic questionnaire and a purpose‐built evaluation grid. This grid was used to evaluate all translated items for their cultural, semantic, conceptual and idiomatic equivalence. Each item was assessed using a Likert scale from 1 to 5 (1—No equivalence; 2—Low equivalence; 3—Some equivalence; 4—High equivalence; 5—Total equivalence).

### 4.2 Participants

The panel members were selected according to the following inclusion criteria: they had to be SLTs, have experience working with PWA, be trained in SCA, and possess a strong command of English.

### 4.3 Statistical Analysis

To assess the content validity of the HPAQ‐EP, a quantitative approach using the Content Validity Index (CVI) was employed. To calculate the CVI for each instrument item, the number of responses with scores of 4 and 5 was summed up and divided by the total number of responses. An acceptable CVI among the experts should be at least 0.80, and preferably higher than 0.90 (Fortin 2009). A descriptive qualitative analysis was also conducted, in which the first and last authors independently reviewed experts’ comments and summarised recurring suggestions and issues through discussion and consensus.

### 4.4 Results

The expert panel responsible for evaluating the consensus version of the HPAQ‐EP consisted of 6 SLTs. All of them were of Portuguese nationality, aged 26–41, with varying academic degrees (from bachelor's–doctorate) and an average of 9 years of experience in aphasia.

During the first round of evaluation, only items 2.1. of the evaluation grid (HPAQ Item 1—How well do you think you understand what aphasia is—idiomatic and conceptual equivalence) and 5 (HPAQ Practice domain—semantic equivalence) resulted in a CVI of 0.70. Since a CVI value of ≥ 0.80, as recommended in the literature (Hoffmann et al. 2014), was not achieved for all questionnaire items and their corresponding equivalences, the items in question were modified based on the expert panel's suggestions. The main suggestions for item modification focused on ensuring uniformity of terminology, improving grammatical accuracy and punctuation and making minor changes to achieve semantic equivalence.

Items whose evaluation was on the margin of approval (CVI = 0.80) were also reviewed, including evaluation grid items 1 (HPAQ Introduction), 2.1 (HPAQ Item 1—How well do you think you understand what aphasia is), 3 (Skills), 3.4 (HPAQ Item 7—If communication with a person with aphasia is unsuccessful I have some strategies to end the conversation in a good way for the person), 4 (HPAQ Attitudes and emotions domain), 4.3 (HPAQ item 10—I experience the situation as frustrating if communication with PWA is unsuccessful), 5 (HPAQ Practice domain), 5.1 (HPAQ item 11—In my daily work with PWA I communicate about complex topics to the same extent as when communicating with people without aphasia), 6 (HPAQ Environment domain) and 6.2 (HPAQ item 14—At my workplace there are materials readily available for me to support communication with PWA).

The same panel carried out a second round of evaluation. The rating grid used in the first round was updated for the items mentioned, and the updated grid was presented to the experts for re‐evaluation and scoring. As a result, a CVI of 1 was achieved for all items, except for items 4.3 (HPAQ item 10—I experience the situation as frustrating if communication with PWA is unsuccessful) and 5.1 (HPAQ item 11—In my daily work with PWA I communicate about complex topics to the same extent as when communicating with people without aphasia) in terms of semantic equivalence, which retained a CVI of 0.80. Thus, the instrument's content validation was concluded.

## 5 Phase 2: Implementation of the *Communicative Training in Aphasia* Program

### 5.1 Procedures

The lead researcher contacted the participants who agreed to take part in the study. The study was explained to them, and they were asked to complete the identification and demographic characterisation sheet and the HPAQ‐EP questionnaire. The lead researcher was present during the completion of the HPAQ‐EP to ensure it was completed without any external assistance. The next step was to assess the HCPs' availability to participate in the training. Two dates were established based on the number of HCPs expected and the desired dynamics.

### 5.2 Participants

The HCPs (Physiotherapists, Nurses, Assistants, Psychologists, Doctors, SLTs and Social Workers) who made up the Clinical Centre team (*N* = 43) were invited to participate in this second phase. The following inclusion criteria were established: being a healthcare professional at the Clinical Centre and having contact with PWA. No exclusion criteria were defined.

### 5.3 Communicative Training in Aphasia

The training was developed by the research team based on the SCA approach and consists of a brief, group‐based communicative training programme for HCPs. The lead researcher delivered it. The programme combined theoretical instruction with practical activities and was evaluated using the HPAQ‐EP to assess changes in HCPs’ knowledge, attitudes and communication strategies when interacting with PWA. It took place in the Clinical Centre auditorium and lasted 2 h. The design and reporting of the study's intervention components were guided by the 12‐item TIDieR checklist, which ensures that interventions in clinical and behavioural studies are described in sufficient detail for replication (Hoffmann et al. 2014). These components are outlined below (Table 1).

**TABLE 1:** Description of the communicative training using the TIDieR framework.

Brief name	Communicative training in aphasia (adapted from SCA (Kagan, 1998)
Why	—PWA are more likely to experience negative outcomes in hospitals compared with patients without aphasia (Bartlett et al. 2008). —HCPs generally have a basic understanding of aphasia, but they frequently lack structured, formal training in effective communication strategies for interacting with PWA (Shrubsole et al. 2021) and frequently choose to communicate with family members instead of directly with the patients themselves (Burns et al. 2015). —Successful communication between PWA and HCPs leads to higher quality medical care, higher patient compliance, increased patient satisfaction and better outcomes (Simmons‐Mackie 2018). –Receiving training in supporting PWA to communicate more effectively (and in communicating more effectively with them) can lead to more successful information transfer and enhanced participation of PWA in their healthcare process (Power et al. 2024).
What (materials and procedures)	—Regarding the theoretical component of the training, the content followed the guidelines proposed by the Aphasia Institute, with a particular focus on the SCA approach, as translated and adapted by the Portuguese Institute of Aphasia (IPA)—a non‐profit organization committed to reducing the psychosocial impact of aphasia, and to prioritizing individuals with aphasia in decision‐making and solution development (Valente 2015). —Key concepts such as communication, language and speech were addressed. In the discussion of communication, its definition was presented, along with the basic skills necessary to optimise it. —The relationship between the quality of communication and the effectiveness and efficiency of healthcare provision was also emphasised. —A clear distinction was made between language and speech to help HCPs understand the difference between interacting with someone with a speech disorder versus someone with a language disorder. This distinction aimed to deepen their understanding of the respective implications for communication. —Additionally, the Portuguese version of the bedside language screening test was introduced as a potential tool to help make this distinction, enabling HPs to adapt their communication strategies based on the results. —Following the clarification of these concepts, the definition of aphasia was explored and examples of aphasic speech were presented. —The emotional impact of aphasia on communication was also discussed, highlighting how the emotional state of the person with aphasia can influence interaction. —The importance of the communication partner's (CP's) attitude was emphasised, focusing on the values and skills that contribute to a supportive and effective communicative relationship. —The next part of the training focused on SCA, a communicative method that includes a set of strategies aimed at facilitating conversation between CPs and PWA. This method was the central focus of the training, as it helps to overcome many of the communication barriers typically encountered. The core pillars of this approach—acknowledging and revealing the communicative competence of the PWA—were discussed, drawing on the work of Kagan et al. (2001; Kagan et al. 2004). —The training concluded with a discussion on the use of augmentative and alternative communication (AAC), emphasising key resources that play a central role in CP‐led interventions. These included supportive attitudes and the use of paper and pencil, which enable the implementation of techniques proposed by SCA. A communication notebook specifically developed for use with individuals with aphasia was shared, along with an informational leaflet designed to summarise and reinforce the strategies covered during the training. —In the practical component of the training, two videos were presented, each depicting a healthcare professional interacting with a person with aphasia—one with and one without the use of supportive communicative strategies. —Finally, a role‐play activity was conducted to help participants understand what it feels like to have aphasia. Working in pairs, one participant assumed the role of a person with aphasia while the other acted as a healthcare professional. The task involved communicating a message known only to one of them, with restrictions placed on key communication methods (e.g., speech and writing). This exercise encouraged the use of alternative forms of communication such as gestures, drawings and images.
Who Provided	The training was prepared and delivered by the lead researcher, with the support of the other authors of the article (AA and AM), two SLTs with more than 20 years of experience working with PWA. AM have received formal training in SCA at the Aphasia Institute in Canada.
How (mode of delivery; individual or group)	Both components of the training were delivered in person and conducted in a group setting.
Where	The training took place in the auditorium of a Clinical Centre.
When and how much	The full training course lasted 2 h, with the practical component comprising a 30 min session.
Tailoring	No adaptations or tailoring were made to the content or format of the Communicative training in Aphasia.
Modifications	No adaptations or modifications were made to the content or format of the Communicative training in Aphasia.
How well	Applying the questionnaires proved challenging due to the dynamics of the service and, at times, the lack of adherence from the HPs. While 43 HCPs were initially involved, only 23 were able to complete the study by its conclusion.

### 5.4 Data Collection Instrument

The HPAQ was developed and validated in Denmark and applied to HCPs before and after communicative training (Jensen et al. 2022). The Intraclass Correlation Coefficient (ICC) for individual items ranged from 0.80 to 0.84, while the overall test‐retest reliability of the HPAQ was excellent (ICC = 0.86). It consists of 16 items that allow the collection of self‐reported scores provided by HCPs in the different aspects that make up the questionnaire, namely Knowledge (i.e., basic information about aphasia and communication), Skills (i.e., communication strategies), Attitudes and Emotions (i.e., their personal opinions or feelings associated with the inability to express oneself), Practice (i.e., implementation of communication skills) and Environment (supportive or non‐supportive role of the work) (Jensen et al. 2022). The scoring method used a blank scale ranging from 0 to 100, where 0 represented the minimum possible rating and 100 the maximum possible rating, on which the HCPs marked the desired area. Subsequently, the marking was measured manually with a ruler and converted into the corresponding value (Jensen et al. 2022).

### 5.5 Statistical Analysis

The statistical data obtained in Phase 2 were processed using the Statistical Package for the Social Sciences (SPSS) version 27.0. Since the HPAQ is scored on a 10 cm visual analogue scale, it was necessary to measure it with a ruler and convert all scores to a 0–100 scale before entering them into the software.

Each participant was identified and matched on the platform using a 6‐digit anonymity code. Corresponding qualitative variables were created to input all demographic, academic and professional information. Based on this, absolute and relative frequencies were calculated for qualitative (sociodemographic) variables and means, and standard deviations were computed for quantitative variables (the scores of the 16 HPAQ items). Internal consistency, reflecting the interrelatedness among items in the scale and assuming unidimensionality, was assessed using Cronbach's *α*. It was calculated for the total scale and subscales and considered adequate if it ranged from 0.70 to 0.95.

The effect of the *Communicative Training in Aphasia* was evaluated by comparing pre‐ and post‐training scores from the HPAQ‐EP. A paired t‐test was used to assess differences between the two time points (pre‐ and post‐training scores). The assumption of normal distribution of the differences between the two time points was verified. For statistically significant results, effect sizes were calculated using Cohen's d.

For all analyses, the significance level was set at < 0.05. Since this is an exploratory study, no power calculation was performed.

### 5.6 Results

The pre‐training sample of participants (the HCPs who agreed to participate in the study, *n* = 39) and those who completed the training (the HCPs who completed all the required steps, *n* = 23) are presented in Table 2.

**TABLE 2:** Sociodemographic characteristics of healthcare professionals.

	Pre‐training (*n* = 39)	Complete training (*n* = 23)
Qualitative variables	*N* (%)	*N* (%)
Gender		
Female	22 (56.4)	15 (65.2)
Male	17 (43.6)	8 (34.8)
Age		
<20	2 (5.1)	1 (4.3)
20–30	4 (10.3)	2 (8.7)
30–40	5 (12.8)	2 (8.7)
40–50	15 (38.5)	10 (43.5)
>50	13 (33.3)	8 (34.8)
Profession		
Nurse	6 (15.4)	2 (8.7)
Nurse assistant	8 (20.5)	2 (8.7)
Physiotherapist	6 (15.4)	6 (26.1)
Psychologist	13 (33.3)	9 (39.1)
Others (social assistant, speech therapist, intern)	6 (15.4)	4 (17.4)
Educational qualification		
<12th grade	2 (5.1)	1 (4.3)
12th grade	7 (17.9)	2 (8.7)
Bachelor's degree	19 (48.7)	12 (52.2)
Master's degree	11 (28.2)	8 (34.8)
Experience		
<1 year	7 (17.9)	3 (13.0)
1–4 years	3 (7.7)	2 (8.7)
5–15 years	9 (23.1)	5 (21.7)
>15 years	20 (51.3)	13 (56.5)

In both situations, females were the predominant sex (56.4% and 65.2%, respectively). Additionally, older age groups were more prevalent, reflected in longer professional experience: more than half of the participants reported over 15 years of professional experience (51.3% and 56.5%, respectively). Regarding educational qualifications, most HCPs hold a bachelor's degree (48.7% and 52.2%, respectively). In terms of profession, psychologists (33.3% and 39.1%, respectively) and physiotherapists (15.4% and 26.1%, respectively) were the most represented, with the latter group comprising the entire team. Notably, there was limited participation from nurses and assistant nurses in the training completion group (only 8.7% for each group).

In the pre‐training setting (*n* = 39), Cronbach's alpha indicated good internal consistency for the total scale (*α* = 0.912), similar to the value reported in the original study (16). For the subscales, internal consistency was also good: ‘Knowledge’ (*α* = 0.812), ‘Skills’ (*α* = 0.918), ‘Practice’ (*α* = 0.803) and ‘Environment’ (*α* = 0.843). However, the ‘Attitudes and Emotions’ subscale presented an alpha of 0.462, possibly indicating that it reflects more than one underlying construct. No information regarding the subscales’ reliability was provided in the original study.

Analysis of the results of the implementation of the *Communicative Training in Aphasia* program revealed that more than half of the HPAQ‐EP items had p‐values < 0.05, indicating statistically significant improvements for Items 1–9 and Item 14 (Table 3). Significant score increases were observed in items related to ‘Knowledge’, ‘Skills’, ‘Attitudes and Emotions’ (two out of three items) and ‘Environment’ (one out of three items). Items related to ‘Practice’ and the remaining two ‘Environment’ items did not show statistically significant improvements. The effect sizes (ES) for the significant results, calculated using Cohen's d, were considered medium to large, ranging from 0.55 to 1.32.

**TABLE 3:** HPAQ‐EP pre‐ and post‐training results (*n* = 23). Cronbach's *alpha* (16 items) = 0.912.

	Pre‐training	Post‐training	Difference (post‐pre)	
Items	M ± SD	M ± SD	M ± SD	Statistical result
Knowledge (*alpha* = 0.812)				
Item 1—How well do you think you understand what aphasia is? (*n* = 21)	64.9 ± 26.2	85.3 ± 11.6	20.4 ± 32.0	*t*(20) = 2.92; *p* = 0.009 *ES* = 0.64
Item 2—How much knowledge do you have about how to communicate best with PWA? (*n* = 22)	35.3 ± 21.6	76.0 ± 17.5	40.7 ± 27.6	*t*(21) = 6.92; *p* < 0.001 *ES* = 1.48
Item 3—How much knowledge do you have about how PWA experience not being able to communicate? (*n* = 22)	42.6 ± 23.6	73.1 ± 22.4	30.5 ± 33.1	*t*(21) = 4.32; *p* < 0.001 *ES* = 0.92

Skills (*alpha* = 0.918)				
Item 4—If PWA cannot say what they want, I have some strategies to help them express themselves in other ways (*n* = 23)	59.8 ± 23.7	83.8 ± 11.9	24.0 ± 25.5	*t*(22) = 4.52; *p* < 0.001 *ES* = 0.94
Item 5—If PWA do not understand what I am saying, I have some strategies to help their understanding (*n* = 23)	48.6 ± 28.7	78.9 ± 18.3	30.3 ± 23.0	*t*(22) = 6.31; *p* < 0.001 *ES* = 1.32
Item 6—If I am not certain that a person with aphasia and I have understood each other correctly, I have some strategies to check this (*n* = 23)	41.1 ± 27.5	77.8 ± 15.9	36.8 ± 29.6	*t*(22) = 6.00; *p* < 0.001 *ES* = 1.25
Item 7—If communication with a person with aphasia is unsuccessful, I have some strategies to end the conversation in a good way for the person (*n* = 23)	50.4 ± 26.5	78.8 ± 19.6	28.3 ± 30.6	*t*(22) = 4.45; *p* = 0.001 *ES* = 0.93

Attitudes and emotions (*alpha* = 0.462)				
Item 8—I am confident that I can communicate with PWA (*n* = 23)	47.0 ± 25.6	74.6 ± 17.7	27.5 ± 21.6	*t*(22) = 6.09; *p* < 0.001 *ES* = 1.27
Item 9—As a health professional, I have a responsibility to make an extra effort when communicating with PWA (*n* = 23)	86.0 ± 14.0	94.2 ± 9.1	8.1 ± 14.3	*t*(22) = 2.75; *p* = 0.012 *ES* = 0.57
Item 10—I experience the situation as frustrating if communication with PWA is unsuccessful (*n* = 23)	67.7 ± 31.8	73.7 ± 26.4	6.0 ± 39.6	*t*(22) = 0.73; *p* = 0.474

Practice (*alpha* = 0.803)				
Item 11—In my daily work with PWA, I communicate about complex topics to the same extent as when communicating with people without aphasia (*n* = 22)	33.6 ± 27.7	30.5 ± 27.7	−3.0 ± 31.7	*t*(21) = −0.45; *p* = 0.658
Item 12—In my daily communication with PWA, I always use strategies to ensure we understand each other (*n* = 22)	75.8 ± 22.1	77.4 ± 26.9	1.6 ± 27.9	*t*(21) = 0.28; *p* = 0.786
Item 13—When planning my work, I always take into consideration the communicative problems of PWA (*n* = 22)	79.9 ± 22.6	78.5 ± 25.9	−1.5 ± 24.5	*t*(21) = −0.28; *p* = 0.782

Environment (*alpha* = 0.843)				
Item 14—At my workplace there are materials readily available for me to support communication with PWA (*n* = 22)	55.4 ± 36.7	73.0 ± 28.2	17.6 ± 32.2	*t*(21) = 2.57; *p* = 0.018 *ES* = 0.55
Item 15—At my workplace there are colleagues who will help me with communication with PWA if I need it (*n* = 22)	86.7 ± 24.5	94.1 ± 12.2	7.4 ± 20.2	*t*(21) = 1.70; *p* = 0.103
Item16—At my workplace there is support from my closest management to take special considerations when working with PWA (*n* = 22)	71.5 ± 35.5	77.6 ± 31.0	6.1 ± 26.4	*t*(21) = 1.09; *p* = 0.290

*ES*: effect size measured by Cohen's d.

Participants reported that during the practical exercise, they struggled not only to understand and follow all the established rules but also to convey messages without speech or writing; most found the lack of vocal feedback from their partner—making it hard to know if the message was understood—to be the greatest challenge. To compensate, pairs relied chiefly on gestures, occasionally using images from their phones and, less often, drawings. Despite these difficulties, feedback was overwhelmingly positive, confirming that the exercise effectively simulated the experience of communicating with aphasia.

## 6 Discussion

Cameron et al. emphasise that HCPs must have the appropriate skills to allow the person with aphasia to be an active member in managing their health and rehabilitation (Cameron et al. 2015). This reinforces the need to invest in literacy in this area to improve health outcomes, the individual and the community. This study aimed to analyse the effectiveness of the theoretical‐practical training, the *Communicative Training in Aphasia*, with a multidisciplinary team from a Clinical Centre in Portugal. To this end, it was necessary to translate and validate a data collection instrument into EP, namely the HPAQ (Jensen et al. 2022).

Regarding the translation of the HPAQ, no significant changes were necessary in the consensus version developed by the translators. During the validation process, the instrument demonstrated strong content validity, with CVI scores for nearly all questionnaire items exceeding the recommended threshold of 0.80. These values were obtained following a second round of evaluation conducted by the same panel of experts, after several items had been revised in response to their initial feedback. It is also important to highlight the inclusion of translators and experts with training and experience in SCA, as their expertise was likely instrumental in achieving the observed positive results.

Internal consistency of HPAQ‐EP was high for the total scale (*α* = 0.912) and across most subscales, particularly ‘Knowledge’ and ‘Skills’, confirming the instrument's psychometric robustness. However, the ‘Attitudes and Emotions’ subscale showed a lower Cronbach's alpha (*α* = 0.462), which may suggest the presence of multiple underlying constructs. The relatively small sample size in the present study (*n* = 39), compared to the original validation sample (*n* = 270), may have influenced these results. Smaller samples are more susceptible to variability in reliability estimates, which may partly explain the low internal consistency score observed.

The second phase of the study, which included pre‐ and post‐administration of the HPAQ‐EP surrounding the *Communicative Training in Aphasia* (a theoretical‐practical workshop), yielded statistically significant improvements in the majority of the scale domains. Positive results were observed in pre‐ and post‐training assessments, in items related to ‘Knowledge’ (items 1, 2 and 3), ‘Skills’ (Items 4, 5, 6 and 7), ‘Attitudes and emotions’ (items 8 and 9) and perceptions of the work ‘Environment’ (item 14). These findings reinforce the importance of providing communication training to HCPs, as previously emphasised by several authors (Barros 2016; Cameron et al. 2015, 2017; Carragher et al. 2024; Cruice, 2018; Kaper et al. 2019).

The best results were observed in the ‘Knowledge’ and ‘Skills’ domains of the scale, highlighting the training's effectiveness in improving HCPs' understanding of aphasia and in equipping them with practical communication strategies to facilitate communication with PWA. These results align with previous findings showing increased confidence and improved communicative behaviour among healthcare teams following similar interventions (Cameron et al. 2015; Cruice, 2018).

Additionally, significant improvements were also observed in several items within the ‘Attitudes and emotions’ and ‘Environment’ domains of the HPAQ‐EP. These results underscore the importance of this type of training not just for knowledge expansion but also for positively influencing HCPs' attitudes toward PWA and raising awareness of environmental factors that may facilitate effective communication with this population. Participant feedback on the practical simulation component of the training where traditional communication methods were restricted—described as both challenging and enlightening—suggests that even short‐format interventions can positively influence HCPs competencies, although these findings also highlight the importance of incorporating more extensive practical sessions and real‐world applications, as recommended by other authors (Cameron et al. 2018; Cruice et al. 2018; Kagan et al. 2001).

In contrast, several items in the ‘Practice’ and ‘Environment’ domains did not show statistically significant differences between pre‐ and post‐training assessments. This may reflect the limited opportunity for participants to apply newly acquired strategies in their clinical practice and in actual clinical settings, rather than just in simulation. Additionally, the short interval between the post‐assessment and the training may not have allowed sufficient time for participants to reflect on, integrate and implement the new knowledge into their daily professional routines. To confirm this possible explanation, it would have been useful to conduct a follow‐up assessment sometime after the training. Moreover, contextual factors such as organisational support and resource availability may fall outside the scope of the training content, thereby limiting its immediate impact and further explaining the lack of improvement in these domains.

### 6.1 Study Limitations

Despite these promising results, several limitations must be acknowledged. In Phase 1, the expert panel was relatively small, despite its members’ extensive experience with aphasia and SCA. Including broader stakeholder input (e.g., PWA themselves) could have strengthened the cultural adaptation. In Phase 2, results demonstrated a positive trend, with increasing mean scores indicating improved knowledge and perceived competence among participants. However, high variability and small sample size may have limited statistical power, preventing some items from reaching statistical significance. To contextualise our findings, we compared our sample size to those used in similar studies on communication training for aphasia. For example, Kagan et al. evaluated the effectiveness of SCA training with a sample of 40 volunteers (Kagan et al. 2001), while Cameron et al. assessed outcomes in a group of 28 physiotherapy and occupational therapy students (33). Although our sample (*n* = 23) is smaller than the former, it remains comparable to the latter. Nevertheless, the limited sample size remains a key limitation, potentially affecting the generalizability and robustness of the observed outcomes.

Selection bias may also have influenced the findings, as certain groups may have been more inclined to volunteer. Another limitation was attrition, particularly among nursing staff, which limited subgroup analysis. The absence of long‐term follow‐up to assess retention of knowledge or the translation of skills into clinical practice represents another limitation of this study. Reassessing the participating HCPs at a later stage would help determine whether the concepts and strategies acquired during the training were retained and effectively integrated into their professional routines.

Another limitation concerns the practical component of the training: its effectiveness might have been greater if it had been conducted directly with PWA, potentially boosting HCPs confidence in applying strategies in real‐world settings. Additionally, using a blank scale for scoring may have limited the accuracy of participants’ responses; future iterations should consider digitalising the scale to minimise measurement error.

Finally, since the HPAQ was published only in 2022, few studies have used it, making comparative discussion more challenging. In fact, the only study we found was by Charalambous et al. (2025), which had a different aim: to explore Cypriot HCPs' knowledge and skills in communicating with PWA, rather than to measure the efficacy of any CPT program.

Future studies should address these limitations by using larger, more diverse samples, adopting longitudinal designs and including PWA in both training and evaluation processes.

### 6.2 Clinical Implications

The findings support integrating targeted communication training into routine professional development for HCPs who interact with PWA. By improving HCPs' knowledge, confidence and communicative strategies, such training fosters more inclusive, patient‐centred care and reduces the risk of marginalisation and medical errors. The validated HPAQ‐EP enables ongoing evaluation of training impact in Portuguese‐speaking settings. Broader implementation and long‐term follow‐up are recommended to maximise benefits and promote sustainable changes in clinical practice. This study advocates for the inclusion of aphasia‐specific communication modules in healthcare curricula to better prepare future HCPs.

## 7 Conclusions

This study detailed the process of translating and validating the content of the HPAQ scale into EP, as well as its application before and after a theoretical‐practical training on communication strategies for aphasia. The training was implemented with a multidisciplinary team of HCPs at a Clinical Centre in Portugal.

Overall, the study's objectives were achieved, as demonstrated by the high CVI values obtained during the translation and validation phases of the HPAQ‐EP version. Additionally, a statistically significant improvement in HCPs' knowledge across various HPAQ dimensions was observed following the training, confirming the intervention's effectiveness.

Future research should consider replicating this study with different HCPs teams across the country. Investigating the long‐term impact of such training on professionals’ ability to support PWA would also offer valuable insights. Integrating PWA into the training assessment, for example, through cognitive interviews, content validity ratings and patient‐reported outcome measures, ensures that the adapted HPAQ‐EP reflects their lived experiences, strengthens its ecological validity and captures their perspectives, thereby enhancing the workshop's real‐world relevance.

Furthermore, the integration of an aphasia‐focused communication module into healthcare education curricula is strongly recommended. Preparing future HCPs with practical tools and evidence‐based strategies would enhance their capacity to engage more effectively with PWA, meet the needs of this vulnerable population, and contribute to a more inclusive and supportive healthcare environment. Mandatory training in this area could significantly improve the quality of care, enhance patient experiences and reduce the stress and frustration often associated with communication barriers.

## Disclosure

The free version of ChatGPT was used to support English language editing.

## Ethics Statement

This research was conducted in accordance with the ethical standards of the Portuguese National Republican Guard (PGNR) Clinical Center in Lisbon and relevant national and international guidelines. All participants involved in this study provided informed consent, and their privacy and confidentiality were strictly protected. No deception was used, and participants retained the right to withdraw at any stage without consequence.

## Conflicts of Interest

The authors report no conflicts of interest.

## Data Availability

The authors affirm that the data was collected, analysed and reported with honesty and transparency.
